# Novel morphology changes from 3D ordered macroporous structure to V_2_O_5_ nanofiber grassland and its application in electrochromism

**DOI:** 10.1038/srep16864

**Published:** 2015-11-18

**Authors:** Zhongqiu Tong, Haiming Lv, Xiang Zhang, Haowei Yang, Yanlong Tian, Na Li, Jiupeng Zhao, Yao Li

**Affiliations:** 1 Center for Composite Materials and Structure, Harbin Institute of Technology, Harbin, 150001, China; 2 School of Chemical Engineering and Technology, Harbin Institute of Technology, Harbin, 150001, China

## Abstract

Because vanadium pentoxide (V_2_O_5_) is the only oxide that shows both anodic and cathodic coloration electrochromism, the reversible lithium ion insertion/extraction processes in V_2_O_5_ lead to not only reversible optical parameter changes but also multicolor changes for esthetics. Because of the outstanding electrochemical properties of V_2_O_5_ nanofibers, they show great potential to enhance V_2_O_5_ electrochromism. However, the development and practical application of V_2_O_5_ nanofibers are still lacking, because traditional preparation approaches have several drawbacks, such as multiple processing steps, unsatisfactory electrical contact with the substrate, expensive equipment, and rigorous experimental conditions. Herein, we first report a novel and convenient strategy to prepare grass-like nanofiber-stacked V_2_O_5_ films by a simple annealing treatment of an amorphous, three-dimensionally ordered macroporous vanadia film. The V_2_O_5_ nanofiber grassland exhibits promising transmittance modulation, fast switching responses, and high color contrast because of the outstanding electrochemical properties of V_2_O_5_ nanofibers as well as the high Li-ion diffusion coefficients and good electrical contact with the substrate. Moreover, the morphology transformation mechanism is investigated in detail.

Electrochromic (EC) materials are characterized by the ability to change their color or optical parameters reversibly and persistently when a voltage is applied across them^1^,^2^,^3^,^4^,^5^. EC devices, especially “smart windows”, have attracted much attention in recent decades because of their promising potential to save energy indoors by controlling solar light transmission through reversible transmittance modulation, as well as the comfortable esthetics accompanying the color changes^1^,^2^,^3^,^4^,^5^. Because of the intrinsic layered lattice anisotropy and the ability of the vanadium ion to change its oxidation state, V_2_O_5_ has shown promising performance in EC applications^6^,^7^,^8^. It is the only oxide that can show both anodic and cathodic coloration, and thus the reversible lithium ion insertion/extraction processes in V_2_O_5_ lead to not only reversible changes in optical parameters but also multicolor changes for esthetics in the voltage range of ±1 V^6^,^7^,^8^,^9^,^10^,^11^. In vanadia EC, the switching speed is limited by the diffusion rate of Li ions during redox reaction processes, while the optical modulation is limited by the number of intercalation sites^8^,^9^,^10^. The relatively small surface area and long Li-ion diffusion distance of conventional flat EC films lead them to exhibit low modulation and slow switching response^10^,^12^. In addition, the electric conductivity (10^−3^ – 0^−2^ S/cm) and Li^+^ diffusion coefficient (10^−13^ – 10^−12^ cm^2^/s) of bulk V_2_O_5_ are both small, and thus the Li-ion intercalation/removal processes are relatively slow^13^,^14^.

One-dimensional (1D) nanofibers have rapidly emerged as the most promising candidates for building films for EC devices^15^,^16^,^17^,^18^,^19^,^20^,^21^,^22^,^23^. The intrinsic advantages of nanofibers for Li-ion intercalation/removal and diffusion kinetics, such as large surface area, facile strain relaxation, and short Li-ion diffusion distance, can significantly enhance the EC performance of oxides^20^. In addition, compared with bulk V_2_O_5_, single-crystal V_2_O_5_ nanofibers have a higher electric conductivity (*ca.* 0.5 S cm^−1^), which can significantly accelerate the redox reaction kinetics^24^,^25^. Furthermore, nanofiber films usually have a porous structure, resulting in interconnected voids for effective electrolyte penetration^15^,^16^,^17^,^18^,^19^,^20^. However, the development and practical application of 1D nanofibers for EC devices are seriously restricted by the techniques used to integrate nanofibers into films. One widely used technique that begins with hydrothermally prepared nanofibers includes hydrothermal preparation, filtration of nanofibers into films and then transfer of the films to transparent current collectors for subsequent testing or usage^15^,^16^,^17^,^18^,^19^,^20^. The multiple processing steps are rather complex for the fabrication of a full EC device and usually lead to weak electrical contact between the filtered films and current collectors, resulting in unsatisfactory switching speeds^15^,^16^,^17^,^18^,^19^,^20^. The vapor transport method^21^,^22^ and catalytic growth process^23^ could generate V_2_O_5_ nanowire arrays or nanoribbon films directly on silicon substrates. However, these approaches usually require expensive equipment and rigorous experimental conditions, and the relatively high preparation temperature (above 600 °C) prevents transparent current collectors from being suitable as alternative substrates to some extent. Therefore, it is a great challenge to develop new techniques to build nanofiber V_2_O_5_ films with enhanced EC performance.

Herein, we propose a novel and convenient strategy to prepare a grass-like nanofiber V_2_O_5_ film as a high-performance EC electrode. In this strategy, the V_2_O_5_ nanofiber grassland directly on indium-doped tin oxide substrate (ITO) was prepared by a simple annealing treatment of an amorphous three-dimensionally ordered macroporous (3DOM) vanadia film. Because of its large surface area, short Li-ion diffusion distance, high Li-ion chemical diffusion coefficient, and low charge-transfer resistance, the V_2_O_5_ nanofiber grassland demonstrated high EC performance. In addition, the morphology transformation mechanism was investigated in detail.

## Results and Discussion

**Figure 1:**
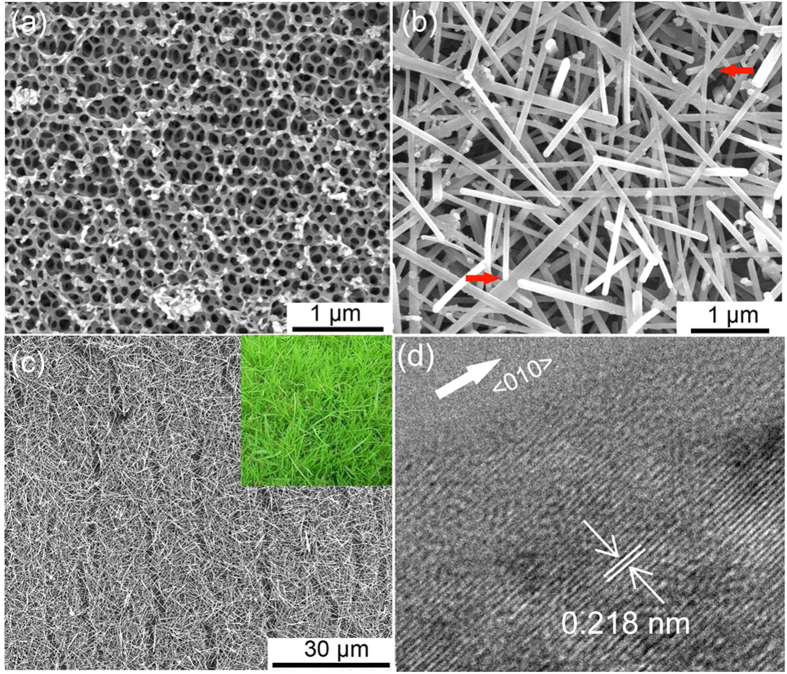
(**a**) SEM image of amorphous 3DOM vanadia film. (**b,c**) SEM images of V_2_O_5_ nanofiber grassland prepared by annealing of amorphous 3DOM vanadia film. The inset picture is a photograph of a real grassland. (**d**) HRTEM image of the tip of a V_2_O_5_ nanofiber. The red arrows in (**b**) show that an elongated nanofiber is composed of two sub-nanofibers attached side-by-side. The white arrow in (**d**) shows the longitudinal direction of the nanofiber.

The 3DOM vanadia films were prepared by anodic deposition of vanadia into polystyrene colloidal crystal templates (described in Methods). Because the nanostructure of the colloidal crystal was replicated in the 3DOM vanadia film, the film exhibited a honeycomb-like structure throughout the entire volume (Fig. 1a and S1). After annealing at 450 °C for 4 h at a rate of 1 °C min^−1^, the 3DOM structure changed completely to a loosely packed nanofiber architecture, which consisted of nanofibers with high length-to-diameter ratios, widths in the range of 40–200 nm, and lengths over 4 μm (Fig. 1b). Interconnected voids and pores formed between the loosely packed nanofibers increased the surface area and were beneficial for electrolyte penetration. Moreover, the loosely packed nanofibers formed a 3D network, allowing fast Li-ion and electron transportation in a 3D manner. As shown in Fig. S2a, a high-magnification scanning electron microscopy (SEM) image shows these nanofibers exhibited smooth surface. Low-magnification SEM images taken of the film indicated that the entire 3DOM structure was morphologically transformed to a grassland-like nanoarchitecture (Fig. 1c). The X-ray diffraction (XRD) pattern of the as-grown nanofibers closely matched that of standard orthorhombic V_2_O_5_ (JCPDS No. 41-1426 with lattice constants *a* = 1.151 Å, *b* = 0.3565 Å, and *c* = 0.4372 Å) (Fig. S2b). Clear lattice fringes along the longitudinal direction of one nanofiber with an interval of approximately 0.218 nm were observed, which corresponded to the *d* spacing of the (002) lattice planes of orthorhombic V_2_O_5_, indicating that the nanofiber grows along the <010> direction (Fig. 1d). The selected area electron diffraction (SAED) pattern taken from the nanofiber showed regular diffraction spots and did not change when the incident electron beam moved along the longitudinal direction of the nanofiber, confirming its single-crystal nature (Fig. S2c). The area of the freestanding V_2_O_5_ nanofiber grassland depended only on the area of the colloidal crystals; thus, the ease of preparation of large-area colloidal crystals suggests that large-scale V_2_O_5_ nanofiber grassland samples are attainable.

It was interesting to find that an elongated nanofiber was composed of two sub-nanofibers attached side-by-side (Fig. 1b, indicated by red arrows). This observation indicated that the coalescence of small adjacent V_2_O_5_ nanorods could play an important role during the elongation growth process of nanofibers. To elucidate the effects of coalescence of adjacent nanosized V_2_O_5_ particles on the formation of V_2_O_5_ nanofibers, the morphological and crystalline changes of 3DOM vanadia films annealed at 450 °C for 0.5 h, 2 h, and 5 h were investigated by SEM, transmission electron microscopy (TEM), and high-resolution TEM (HRTEM).

**Figure 2:**
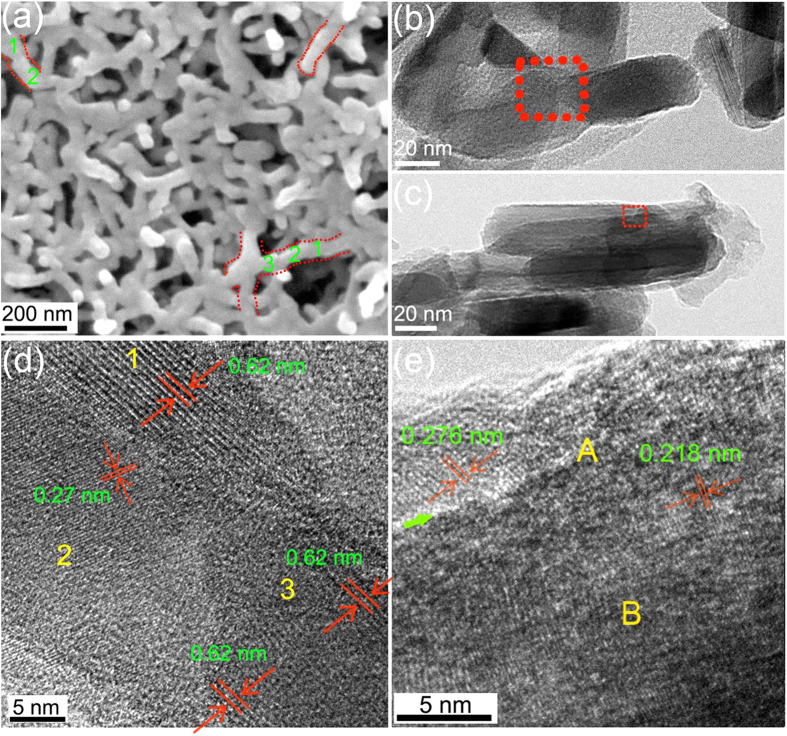
(**a**) SEM image of 3DOM vanadia film annealed at 450 °C for 0.5 h. (**b**) TEM image of three nanoparticles attached together with two nanoparticles standing in a line. (**c**) TEM image of several nanoparticles attached together in a shoulder-to-shoulder manner. (**d**) HRTEM image of the area indicated by the red square in (**b**). (**e**) HRTEM image of the area indicated by the red square in (**c**). The green arrow in (**e**) shows the crystal boundary.

As shown in Fig. 2a, after annealing for 0.5 h, the amorphous 3DOM nanostructure changed into a 3D architecture consisting of nanoparticles. A previous study showed that the surface of electrodeposited amorphous 3DOM vanadia had many V_2_O_5_·1.6H_2_O nuclei. Under thermal activation, these nuclei released water molecules into V_2_O_5_ nanocrystallites. The coalescence of the nanocrystallites and the induced crystallization between the V_2_O_5_ nanocrystallites and the amorphous vanadia caused the disintegration of the 3DOM structure and then morphological transformation into nanoparticles with an amorphous coating layer^26^. Remarkably, many worm-like nanoparticle aggregations were observed. For instance, aggregations formed by the attachment of at least three nanoparticles (marked as 1, 2, and 3) and two nanoparticles (marked as 1 and 2) in a line were indicated in Fig. 2a. TEM and HRTEM were used to further detect the crystallographic relationship of the adjacent nanoparticles in the aggregations. Figure 2b shows three rod-like nanoparticles aggregated together with two nanoparticles standing in a line. An HRTEM image of the interconnecting position clearly showed that the two adjacent nanoparticles standing in a line shared an almost common crystallographic orientation (the two nanocrystallites marked as 1 and 3 in Fig. 2d). A common crystallographic orientation can lead to the easy coalescence of two nanoparticles into an elongated nanorod^27^,^28^,^29^,^30^. In addition, the propensity for atom rearrangement of the amorphous layer on the nanoparticles could increase the effectiveness of the coalescence^26^. Because the nanoparticles were stacked in a 3D network, there must have been an enormous quantity of such nanoparticle aggregations throughout the entire film, leading to the growth of elongated nanorods along various directions. The crystalline lattice changes of the nanoparticle (marked as 2 in Fig. 2d) during the annealing process will be discussed in the following section. It is also important to discuss the changes in the crystallographic structure when two nanoparticles are in a parallel arrangement, because it is closely related to the widening process of the nanorods. Figure 2c illustrates the situation when several rod-like nanoparticles were in shoulder-to-shoulder manner. Clear atom rearrangement can be found at the boundary of the two nanoparticles (marked as “A” in Fig. 2e). In addition, atom rearrangement also occurred in the inner part of the nanoparticle where marked as “B” in Fig. 2e. Atom rearrangement can eliminate grain boundaries and lattice defects to reduce the system energy. A clearer and larger HRTEM image of Fig. 2e is shown in Fig. S3.

**Figure 3:**
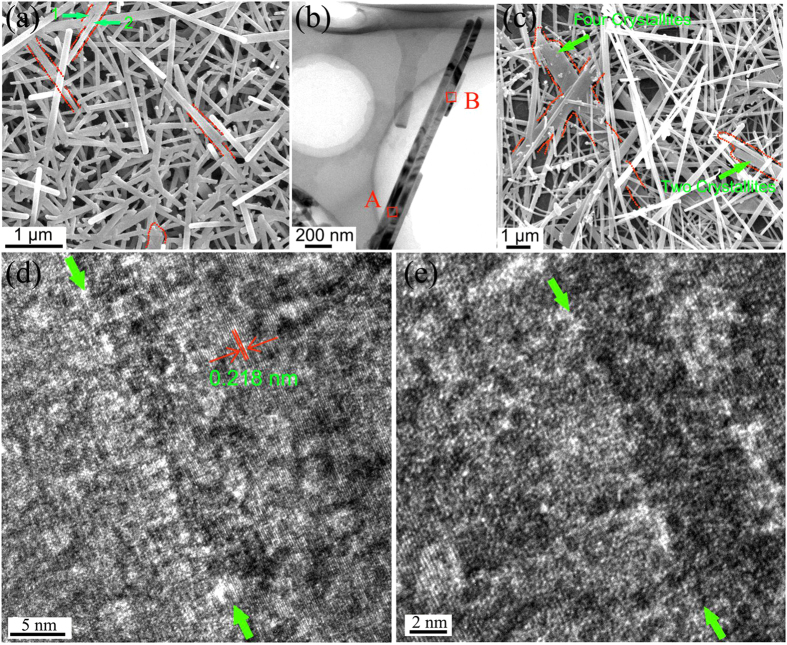
(**a**) SEM image of 3DOM vanadia film annealed at 450 °C for 2 h. (**b**) TEM image of four nanorods attached shoulder to shoulder taken from the sample annealed at 450 °C for 2 h. (**c**) SEM image of 3DOM vanadia film annealed at 450 °C for 5 h. (**d**) HRTEM image of the area indicated by red square “A” in (**b**). (**d**) HRTEM image of the area indicated by red square “B” in (**b**). The green arrows in (**d,e**) show the crystal boundary.

Figure 3a shows the SEM image of the 3DOM film annealed at 450 °C for 2 h. The elongated nanorods along various directions confirmed the existence of enormous worm-like nanorod aggregations along various directions in the early annealing stage (annealing time for 0.5 h) and the coalescence of nanorods along the longitudinal direction. Furthermore, the widening of nanorods confirmed the coalescence of nanorods along the transversal direction. In the high-magnification SEM image of this film (Fig. S4), some nanofibers were decorated with small crystallites. Figure 3a shows an elongated nanorod consisting of two parallel nanorods, and its integrated part had a perfect rod shape. HRTEM was used to investigate the crystallographic relationship of the nanorods when they stacked shoulder to shoulder. As shown in Fig. 3b, four nanorods were attached together shoulder to shoulder. These nanorods were strongly attached to each other even after strong sonication for a long time during the TEM specimen preparation process, implying the strong interaction between these nanorods. The interfaces formed by the stacking of four nanorods provided evidence of transversal coalescence for widening. Detailed HRTEM observations shown in Fig. 3d (at “A”) and 3e (at “B”) demonstrated that the adjacent nanorods nearly had a common crystallographic orientation. An overlap with a common crystallographic orientation is consistent with previous reports of a so-called “oriented attachment” process that can effectively drive adjacent nanorods into a single-crystal nanostructure, because it can substantially reduce the surface energy^27^,^28^,^29^,^30^. This is the first time that an oriented attachment mechanism has been found in a solid-state crystallite coalescence process. Figure S5 shows a nanorod with a single-crystal nanostructure formed by overlapping two nanorods standing shoulder to shoulder, which confirmed the existence of an oriented attachment mechanism during the annealing process. With the annealing time further increasing to 5 h, nanobelts were observed (Fig. 3c). For instance, a nanobelt formed by the parallel attachment of at least four nanorods was observed. The large number of nanobelts found indicates that the “oriented attachment” coalescence mechanism occurred throughout the film. Concomitantly, there was also a significant increase in the length of the nanofibers with an increase in the annealing time from 2 h to 4 h or 5 h. In addition, atom rearrangement between the crystal boundaries could increase the effectiveness of the oriented attachment coalescence. Atom rearrangement also occurred when two crystallites were attached at a wide interfacial angle. Oriented attachment cannot shape the crystallite labeled as “2” in Fig. 2d into a single crystal with another two crystallites marked as “1” and “2” because of the wide interfacial angle. However, an atom rearrangement process could occur to eliminate the grain boundary and reduce the system energy. As shown in Fig. S6, a clear atom rearrangement process was found between two crystallites with a wide interfacial angle in the film annealed for 2 h. In addition, small crystallites decorating the nanofibers (in the film annealed for 2 h) disappeared when the annealing time was increased to 4 h, indicating that Ostwald ripening, referring to a process where larger particles grow at the expense of smaller ones, also occurred during the growth process of the nanofibers^31^,^32^.

**Figure 4:**
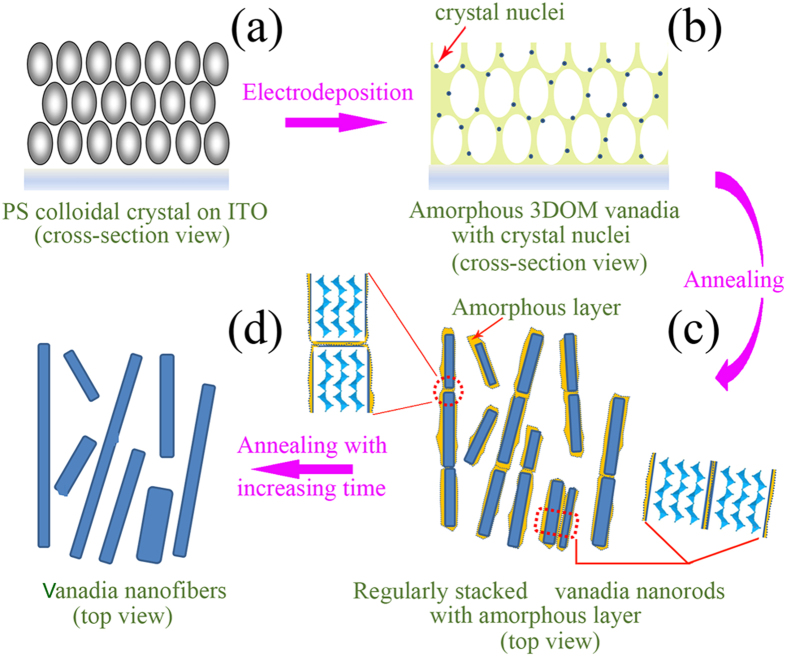
Schematic of synthesis of V_2_O_5_ nanofiber grassland from amorphous 3DOM vanadia film. (**a**) PS colloidal crystal template. (**b**) Electrodeposited amorphous 3DOM vanadia film. (**c**) Change in morphology of amorphous 3DOM vanadia into nanorods with amorphous layers via annealing. (**d**) Coalescence of adjacent nanorods into nanofibers via annealing.

Based on the discussion above, it is reasonable to deduce that the elongation process of nanofibers is also based on the coalescence of adjacent nanorods with similar crystallographic orientation and an atom rearrangement process. During the coalescence of adjacent nanorods for elongation into a nanofiber, if there were an apparent crystallographic orientation difference between the adjacent nanorods, the atoms would not have sufficient time for rearrangement into the elongated nanofiber with a single-crystal nanostructure, and the nanofibers should be crooked. Therefore, a model for the synthesis of V_2_O_5_ nanofibers from the amorphous 3DOM vanadia structure by annealing treatment is proposed (Fig. 4). The surface charge and roughness of the colloidal spheres resulted in heterogeneous nucleation on the surfaces of the colloidal spheres during electrodeposition, leading to the distribution of a large number of V_2_O_5_·1.6H_2_O crystal nuclei on the surface of the amorphous 3DOM skeleton. Under thermal activation, V_2_O_5_·1.6H_2_O nuclei on the surface of the 3DOM walls release water molecules into V_2_O_5_ nanocrystallites^26^. The coalescence of the nanocrystallites and the induced crystallization between the V_2_O_5_ nanocrystallites and the amorphous vanadia causes the disintegration of the 3DOM structure and then growth of nanorods. Because the nanorods are prepared from the 3DOM structure, many nanorods with a similar crystallographic orientation stand in a line, and some nanorods are in a parallel arrangement with a similar crystallographic orientation (Fig. 4a–c). Under further thermal activation, the coalescence of adjacent nanorods along a line leads to elongated nanofibers, while the coalescence of the adjacent nanorods in a parallel arrangement widens the nanofibers. In addition, atom rearrangement and Ostwald ripening also play important roles during the morphological transformation process (Fig. 4d). The crystal growth process of V_2_O_5_ nanfibers from an amorphous 3DOM vanadium oxide film is the synergetic result of atom rearrangement, Ostwald ripening, and oriented attachment mechanisms.

**Figure 5:**
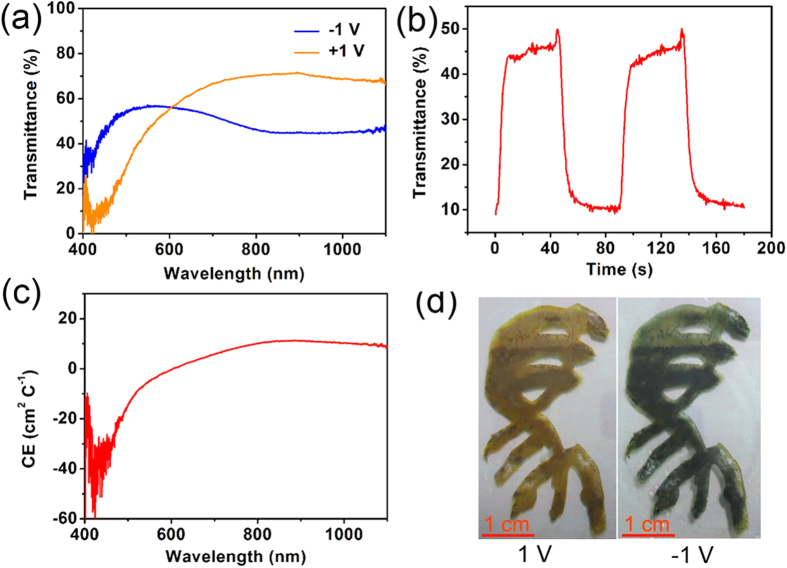
EC performance of V_2_O_5_ nanofiber grassland. (**a**) Transmittance modulation of the colored (+1 V) and bleached (−1 V) states. (**b**) Switching curves for λ = 450 nm under a potential impulse of ±1 V. (**c**) Coloration efficiency. (**d**) EC digital photographs of the colored (+1 V) and bleached (−1 V) states.

In view of the high electrochemical activity of nanofibers and the interconnected voids for effective electrolyte penetration, the V_2_O_5_ nanofiber grassland has great potential for EC properties. Under potentials of +1 V and −1 V (*vs.* Ag/AgCl), the architecture exhibited obvious transmittance modulation, and *ca.* 34% transmittance contrast was obtained when λ = 450 nm. At a near-infrared wavelength of 1000 nm, a transmittance of *ca.* 25% was observed (Fig. 5a). Table S1 shows a comparison of the EC performance of the V_2_O_5_ nanofiber grassland with that of other dense films and nanostructures of vanadia. As displayed in the table, the V_2_O_5_ nanofiber grassland exhibited higher transmittance modulation than many other vanadia nanostructures. Under an alternating potential between −1 V and +1 V, the color-switching time, defined as 90% of the total transmittance change, was about 8.9 s for coloration and 7.4 s for bleaching at 450 nm, as shown in Fig. 5b. These two switching speeds of the V_2_O_5_ nanofiber grassland are much faster than those of many V_2_O_5_ nanofiber films. Takahashi *et al.* reported that a 30% transmittance modulation at 700 nm took 50 s for a V_2_O_5_ nanorod array when 3.0 V was applied, and 300 s was required for a sol-gel V_2_O_5_ film^7^. Xiong *et al.* reported the color-switching time of a filtered V_2_O_5_ nanowire film to be *ca.* 12.5 s for coloration under −1.5 V and *ca.* 10.4 s for bleaching under +2.5 V^15^. Cheng *et al.* showed that the V_2_O_5_ nanowires grown on an ITO substrate exhibited *ca.* 13.9 s and 10.8 s for coloration and bleaching, respectively^21^.

Another important criterion for EC materials is the coloration efficiency (*CE*), which is defined as the change in optical density (*OD*) divided by the inserted charge per unit area (*Q*_in_), as expressed in the following equations:


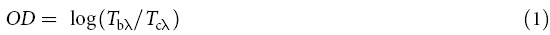



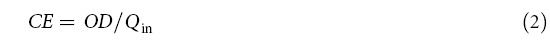


where *T*_bλ_ and *T*_cλ_ represent the transmittance of the bleached and colored samples, respectively. Obviously, *CE* represents the ability of optical modulation during the coloration-bleaching process while considering energy consumption. As shown in Fig. 5c, the nanofiber grassland showed larger *CE* values in the spectral range of 400–500 nm, because the color changes occurred in this range. At a wavelength of 450 nm, a high *CE* value of 32 cm^2^ C^−1^ was obtained. In the NIR range, an average CE value of 8 cm^2^ C^−1^ was obtained.

A large-area EC film was prepared with large-scale colloidal crystals as a template (Fig. 5d). Lithium intercalation into V_2_O_5_ under a cathodic potential of −1 V caused a homogeneous transmission of *ca*. 50% across the entire visible range, resulting in a dark green coloration. Charge extraction under an anodic potential of +1 V caused strong absorption in the 400–500 nm spectral range, leading to a yellow color.

**Figure 6:**
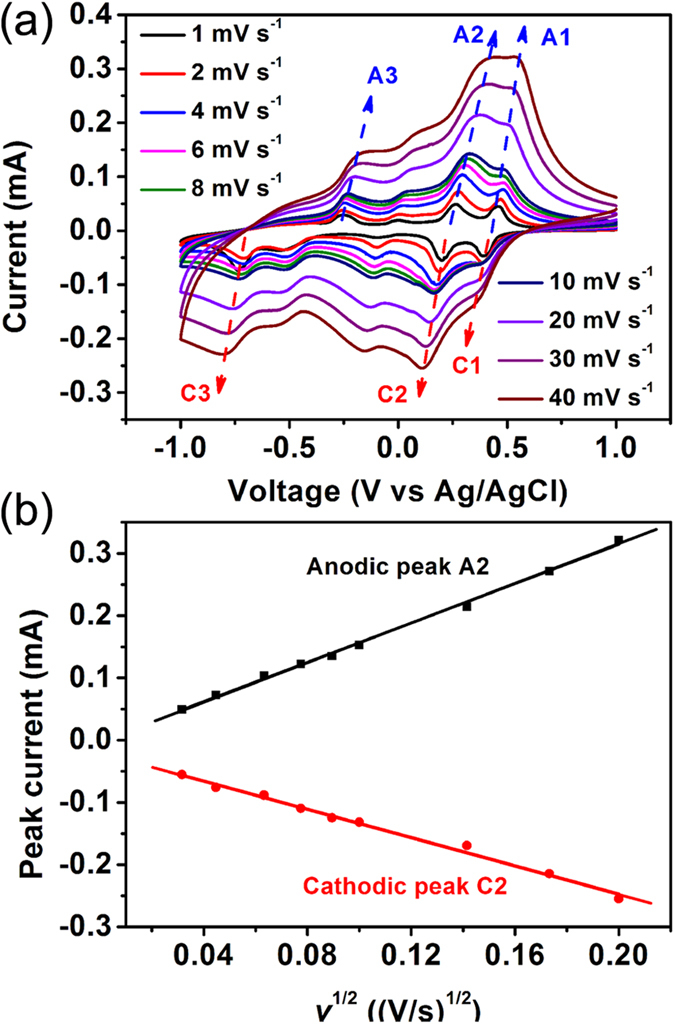
(**a**) CV curves of V_2_O_5_ nanofiber grassland at various scan rates. (**b**) Fitting plots between the peak current *i* and the square root of the scan rate *v*^1/2^.

To further evaluate the Li-ion diffusion coefficient in the V_2_O_5_ nanofibers, cyclic voltammetry (CV) was performed on the free-standing V_2_O_5_ nanofiber grassland with different potential scanning rates in the potential range of ±1 V (*vs.* Ag/AgCl). As shown in Fig. 6a, the film showed a typical electrochemical CV curve of orthorhombic V_2_O_5_ materials^6^,^11^. The three cathodic peaks located at 0.38 (marked as C1), 0.19 (marked as C2), and –0.70 V (marked as C3) correspond to α/*ε*, *ε*/*δ*, and *δ*/*γ* phase transitions, respectively, according to the literature. The three anodic peaks, marked as A1, A2, and A3, can be attributed to the corresponding *ε*/*α*, *δ*/*ε*, and *γ*/*δ* phase transitions, respectively. The reduction peaks shifted to lower potentials while the oxidation peaks moved to higher ones with an increase in the scan rate. At low scan rates, all the active surface area can be utilized for redox reactions. However, at high scan rates, diffusion limits the movement of Li^+^ ions because of the time constraint, and only the outer active surface is utilized for redox reactions, thus leading to electrode polarization, which results in the shifts of the redox peaks^12^,^15^. The appearance of redox peaks even at a high scan rate of 0.4 V s^−1^ indicated that the redox reactions in the V_2_O_5_ nanofiber grassland were reversible. For a simple solid-state diffusion-controlled process, the effective diffusion coefficient can be estimated from the Randles-Servcik formula^5^,^33^:


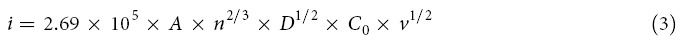


where *i*, *D*, and *v* represent the peak current, effective diffusion coefficient, and potential scan rate, respectively; *A*, *n*, and *C*_0_ represent the effective surface area of the electrode, number of electrons transferred in the unit reaction, and concentration of diffusion species (Li ions), respectively. To reasonably characterize the Li^+^ diffusion coefficients during the Li-ion insertion and extraction processes, the redox couple between the *ε* and *δ* phase transitions (A2 and C2) was chosen for calculation.

Figure 6b shows the correlations between the peak current *i* and the square root of the scan rate *v*^1/2^ for the A2 and C2 peaks. The good linear relationship demonstrated that the oxidation/reduction processes of V_2_O_5_ nanofibers were controlled by ion diffusion from the electrolyte to the electrode surface. As calculated using Equation (3), the Li^+^ ion chemical diffusion coefficients for the film were 1.8 × 10^−11^ cm^2^ s^−1^ and 4.6 × 10^−10^ cm^2^ s^−1^ for A2 and C2, respectively. The Li^+^ diffusion coefficients of this V_2_O_5_ nanofiber grassland were relatively high compared with the corresponding values in the literature. Xiong *et al.* reported that the lithium diffusion coefficient was 5.3 × 10^−10^ cm^2^ s^−1^ in a Ag_0.35_V_2_O_5_ hydrothermal nanowire stacked porous film and 7.5 × 10^−11^ cm^2^ s^−1^ in a V_2_O_5_ hydrothermal nanowire stacked porous film^15^. McGraw *et al.* reported that the maximum diffusion coefficient was 1.7 × 10^−12^ cm^2^ s^−1^ in a V_2_O_5_ film prepared by pulsed laser deposition^14^. Julien *et al.* reported a value of ~10^−12^ cm^2^ s^−1^ for flash-evaporated V_2_O_5_ films^34^.

**Figure 7:**
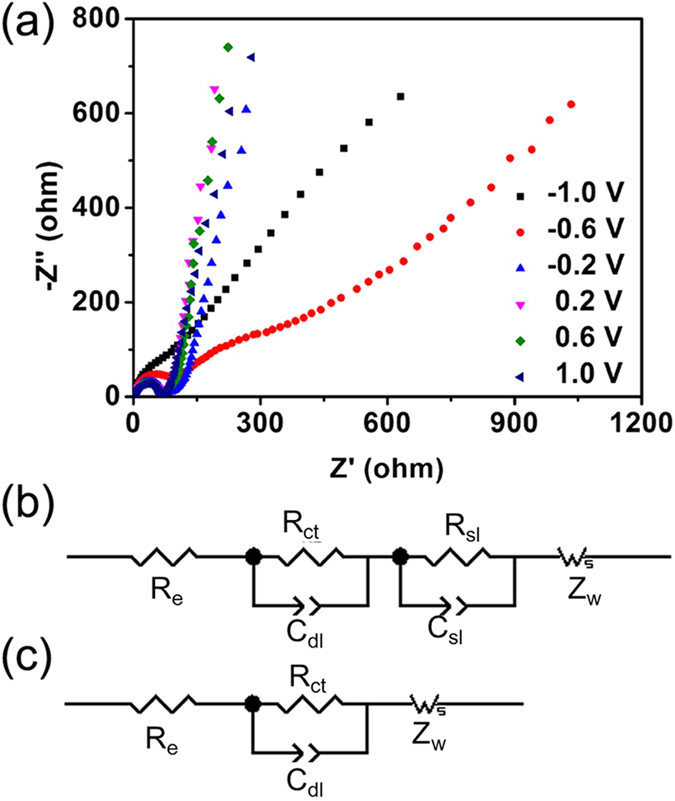
(**a**) Nyquist diagrams of the V_2_O_5_ nanofiber grassland acquired within the range of ±1 V with 0.4 V potential intervals. (**b**) The equivalent circuit for the film under potentials from 1 V to −0.2 V. (**c**) The equivalent circuit for the film under potentials −0.6 V to −1 V.

Electrochemical impedance spectroscopy (EIS) was performed in order to better understand the charge transfer characteristics of the V_2_O_5_ nanofiber grassland during the EC process in a ±1 V potential range (*vs.* Ag/AgCl). Figure 7a displays the Nyquist diagrams acquired for ±1 V with 0.4 V potential intervals. As shown in the figure, the Nyquist diagrams did not change significantly in the potential range between 1 and −0.2 V. At high frequency, a well-defined semi-circle was always observed with a similar shape and magnitude. This semi-circle can be ascribed to the charge transfer resistance (*R*_ct_). The straight line with a phase angle of around 45 ° from the real axis corresponds to the Warburg impedance (*Z*_w_). At low frequencies, the capacitive line was related to the finite diffusion process. The corresponding simulated circle is shown in Fig. 7c. *R*_e_ corresponded to the total resistance, which combined the resistance of the electrolyte and the resistance between the V_2_O_5_ nanofibers and the ITO substrate. *C*_dl_ represented the double-layer capacitance. When the polarized potentials were −0.6 V and −1 V, the film exhibited another semi-circle between the frequencies represented by the Warburg impedance and the frequencies represents by the charge transfer resistance. The corresponding simulated circle is shown in Fig. 7b. *R*_sl_ and *C*_sl_ denoted the resistance to the migration of Li ions and the capacity of the layer, respectively. The simulated *R*_e_ and *R*_ct_ values of the V_2_O_5_ nanofiber grassland under different polarized potentials are listed in Table S2. As shown in the table, the nanofiber grassland exhibited a low *R*_e_ of less than 8.4 Ω over the entire potential range, suggesting good electrical contact between the nanofibers and the ITO substrate and good electrolyte penetration into the V_2_O_5_ nanofiber grassland, which can facilitate the redox reaction process, improving the switching response speed. Because of the high electrical conductivity of V_2_O_5_ nanofibers, the architecture also exhibited low charge transfer resistance (*R*_ct_). Under a potential of 1 V, the nanofiber grassland exhibited a low *R*_ct_ value of 59.1 Ω, which increased slightly to 68.7 Ω when *δ*-V_2_O_5_ was obtained under −0.2 V. When the potential was −0.6 V where the phase transition from *δ*-V_2_O_5_ to *γ*-V_2_O_5_ occurred, the decreased electrical conductivity in V_2_O_5_ led the *R*_ct_ value increase to 98.8 Ω, and it finally increased to 136.1 Ω at −1 V. Compared with corresponding values from the literature, our film exhibited very low *R*_ct_ values. Under a potential of 0.2 V, the oriented V_2_O_5_ thin platelets stacked in a porous film exhibited an *R*_ct_ of *ca.* 85 Ω^35^, while *ca.* 200 Ω *R*_ct_ was found for a V_2_O_5_ xerogel film^36^. The Ti-doped vanadium oxide thin compact film exhibited a minimum *R*_ct_ of *ca.* 578 Ω at −0.6 V^37^. The low charge-transfer-resistance values of the V_2_O_5_ nanofiber grassland could accelerate the double electron-ion injection process, further improving the switching response speed.

## Conclusions

In summary, we have demonstrated a novel strategy to prepare a V_2_O_5_ nanofiber grassland via a simple annealing treatment of an amorphous 3DOM vanadia film. The coalescence of adjacent nanorods with a similar crystallographic orientation, atom rearrangement, and Ostwald ripening were responsible for the growth of nanofibers under thermal activation. The nanofiber grassland displayed promising transmittance modulation (*ca.* 34% at 460 nm and *ca.* 25% at 1000 nm), fast switching responses (8.9 s for coloration and 7.4 s for bleaching), and high color contrast (yellow and dark green) because of the large number of electroactive sites, short Li-ion diffusion distance, high Li-ion chemical diffusion coefficient, and low charge-transfer resistance. The V_2_O_5_ nanofiber grassland presented here could find application not only in EC devices but also in Li-ion batteries, supercapacitors, sensors, catalysts, and other devices. In addition, this synthesis method has demonstrated that it is possible to prepare nanofibers by a facile solid-state approach, which could be extended to other metal oxides with a layered structure.

## Methods

Monodispersed polystyrene (PS) latex spheres (diameters of 250 nm) were obtained using emulsifier-free emulsion polymerization technology^38^. PS colloidal crystal templates were grown using a controlled vertical drying method^38^. ITO (~9 Ω/cm^2^) was cleaned ultrasonically in acetone, methanol, and then distilled water for 20 min each. The cleaned ITO substrates were placed in cylindrical vessels. A PS sphere suspension diluted to 0.5 wt% was added to the glass vessels and then evaporated in an incubator at a stable temperature of 60 °C. An SEM image and digital photograph of a PS colloidal crystal template are shown in Fig. S7.

The anodic deposition of vanadia into polystyrene colloidal crystal templates was performed at a constant voltage of 2 V versus Ag/AgCl from a 1:1 mixture (volume ratio) of distilled water and ethanol containing 0.25 M VOSO_4_·5H_2_O with a Pt foil as the counter electrode. The deposition time was about 40 s. Ethanol was used to reduce the surface tension between the electrolyte and the polystyrene surface. The pH of the electrolyte was adjusted to 2.7 using NaOH.

After deposition, samples were immersed in a 1:1 mixture (volume ratio) of DMF and toluene to remove the PS templates. Finally, the 3DOM samples were annealed at 450 °C for 4 h at a rate of 1 °C min^−1^ in air to obtain the V_2_O_5_ nanofiber grassland. To analyze the morphology transformation mechanism, 3DOM films annealed at different times (0.5 h, 2 h, and 5 h) were also prepared.

XRD was performed to investigate the crystalline structure using an X’Pert PRO X-ray diffractometer with Cu Kα radiation. SEM images were collected with a FEI Helios Nanolab 600i at an acceleration voltage of 20 kV. TEM images, HRTEM images, and SAED patterns were recorded using an FEI Tecnai G2F30. The electrochemical and EC properties were measured with a three-electron system (CHI 660C electrochemical workstation) with a 1 M solution of LiClO_4_ in propylene carbonate (PC) as the electrolyte. A platinum plate acted as the counter electrode, an Ag/AgCl electrode acted as the reference electrode, and an ITO substrate coated with V_2_O_5_ acted as the working electrode. *In situ* visible and near-infrared EC measurements were performed using an optic spectrometer (MAYA 2000-Pro, Ocean Optics).

## Additional Information

**How to cite this article**: Tong, Z. *et al.* Novel morphology changes from 3D ordered macroporous structure to V_2_O_5_ nanofiber grassland and its application in electrochromism. *Sci. Rep.* **5**, 16864; doi: 10.1038/srep16864 (2015).

## Supplementary Material

Supplementary Information

## References

[b1] Rosseinsky D. R. & Mortimer R. J. Electrochromic systems and the prospects for devices. Adv. Mater. 13, 783–793 (2001).

[b2] Granqvist C. G. Oxide electrochromics: An introduction to devices and materials. Sol. Energy Mater. Sol. Cells 99, 1–13 (2011).

[b3] Malgras V. *et al.* Templated Synthesis for Nanoarchitectured Porous Materials. Bull. Chem. Soc. Jpn. doi: 10.1246/bcsj.20150143 (2015).

[b4] Niklasson G. A. & Granqvist C. G. Electrochromics for smart windows: thin films of tungsten oxide and nickel oxide, and devices based on these. J. Mater. Chem. 17, 127–156 (2007).

[b5] Dai S. G. *et al.* MnO_2_@KCu_7_S_4_ NWs hybrid compositions for high-power all-solid-state supercapacitor. J. Power Sources 274, 477 (2015).

[b6] Chernova N. A., Roppolo M., Dillon A. C. & Whittingham M. S. Layered vanadium and molybdenum oxides: batteries and electrochromics. J. Mater. Chem. 19, 2526–2552 (2009).

[b7] Takahashi K., Wang Y. & Cao G. Z. Growth and electrochromic properties of single-crystal V_2_O_5_ nanorod arrays. Appl. Phys. Lett. 86, 053102 (2005).

[b8] Scherer M. R. J., Li L., Cunha P. M. S., Scherman O. A. & Steiner U. Enhanced Electrochromism in Gyroid-Structured Vanadium Pentoxide. Adv. Mater. 24, 1217–1221(2012). 22287187 10.1002/adma.201104272

[b9] Yang Y., Kim D. & Schmuki P. Electrochromic properties of anodically grown mixed V_2_O_5_-TiO_2_ nanotubes. Electrochem. Commun. 13, 1021–1025 (2011).

[b10] Li L., Steiner U. & Mahajan S. Improved electrochromic performance in inverse opal vanadium oxide films. J. Mater. Chem. 20, 7131–7134 (2010).

[b11] Liu Y. Y. *et al.* V_2_O_5_ Nano-Electrodes with High Power and Energy Densities for Thin Film Li-Ion Batteries. Adv. Energy Mater. 1, 194–202 (2011).

[b12] Tong Z. Q. *et al.* Versatile displays based on a 3-dimensionally ordered macroporous vanadium oxide film for advanced electrochromic devices. J. Mater. Chem. C 3, 3159–3166 (2015).

[b13] Portiron E., Salle A. L., Varbaere A., Piffard Y. & Guyomard D. Electrochemically synthesized vanadium oxides as lithium insertion hosts. Electrochim. Acta 45, 197–214 (1999).

[b14] McGraw J. M. *et al.* Li ion diffusion measurements in V_2_O_5_ and Li(Co_1−x_Al_x_)O_2_ thin-film battery cathodes. Electrochim. Acta 45, 187–196 (1999).

[b15] Xiong C. R., Aliev A. E., Gnade B. & Balkus K. J. Fabrication of silver vanadium oxide and V_2_O_5_ nanowires for electrochromics. ACS Nano 2, 293–301 (2008). 19206630 10.1021/nn700261c

[b16] Kang W. *et al.* Green synthesis of nanobelt-membrane hybrid structured vanadium oxide with high electrochromic contrast. J. Mater. Chem. C 2, 4727–4732 (2014).

[b17] Bastakoti B. P. *et al.* Mesoporous carbon incorporated with In_2_O_3_ nanoparticles as high-performance supercapacitors. Eur. J. Inorg. Chem. 2013, 1109–1112 (2013).

[b18] Wang J. M., Khoo E., Lee P. S. & Ma J. Synthesis, assembly, and electrochromic properties of uniform crystalline WO_3_ nanorods. J. Phys. Chem. C 112, 14306–14312 (2008).

[b19] Huang H. S. *et al.* Evaporation-Induced Coating of Hydrous Ruthenium Oxide on Mesoporous Silica Nanoparticles to Develop High-Performance Supercapacitors. Small 9, 2520–2526 (2013). 23494855 10.1002/smll.201202786

[b20] Ellis B. L., Knauth P. & Djenizian T. Three-Dimensional Self-Supported Metal Oxides for Advanced Energy Storage. Adv. Mater. 26, 3368–3397 (2014). 24700719 10.1002/adma.201306126

[b21] Cheng K. C., Chen F. R. & Kai J. J. V_2_O_5_ nanowires as a functional material for electrochromic device. Sol. Energy Mater. Sol. Cells 90, 1156–1165 (2006).

[b22] Chan C. K. *et al.* Fast, completely reversible Li insertion in vanadium pentoxide nanoribbons. Nano Lett. 7, (490–495) 2007. 17256918 10.1021/nl062883j

[b23] Velazquez J. M. & Banerjee S. Catalytic Growth of Single-Crystalline V_2_O_5_ Nanowire Arrays. Small 5, (1025–1029) 2009. 19235798 10.1002/smll.200801278

[b24] Zhai T. Y. *et al.* Centimeter-Long V_2_O_5_ Nanowires: From Synthesis to Field-Emission, Electrochemical, Electrical Transport, and Photoconductive Properties. Adv. Mater. 22, 2547–2552 (2010). 20449845 10.1002/adma.200903586

[b25] Muster J. *et al.* Electrical transport through individual vanadium pentoxide nanowires. Adv. Mater. 12, 420–424 (2000).

[b26] Tong Z. Q. *et al.* From Amorphous Macroporous Film to 3D Crystalline Nanorod Architecture: A New Approach to Obtain High-Performance V_2_O_5_ Electrochromism. Adv. Mater. Interfaces. 2, 1500230 (2015).

[b27] Li M., Kong F. Y., Wang H. Q. & Li G. H. Synthesis of vanadium pentoxide (V_2_O_5_) ultralong nanobelts via an oriented attachment growth mechanism. CrystEngComm. 13, 5317–5320 (2011).

[b28] Penn R. L. & Banfield J. F. Imperfect oriented attachment: dislocation generation in defect-free nanocrystals. Science 281, 969–971 (1998). 9703506 10.1126/science.281.5379.969

[b29] Liu B. *et al.* Morphology control of stolzite microcrystals with high hierarchy in solution. Angew. Chem. Int. Ed. 43, 4745–4750 (2004). 10.1002/anie.200460090 15366075

[b30] Cölfen H. & Antonietti M. Mesocrystals: inorganic superstructures made by highly parallel crystallization and controlled alignment. Angew. Chem. Int. Ed. 44, 5576–5591 (2005). 10.1002/anie.200500496 16035009

[b31] Menon J., Suryanarayana C. & Singh G. Polytypism in a decagonal quasicrystalline Al-Co phase. J. Appl. Cryst. 22, 96–99 (1989).

[b32] Ouyang R., Liu J.-X. & Li W.-X. Atomistic Theory of Ostwald Ripening and Disintegration of Supported Metal Particles under Reaction Conditions. J. Am. Chem. Soc. 135, 1760–1771 (2013). 23272702 10.1021/ja3087054

[b33] Zhang J.-G., Tracy E. C., Benson D. K. & Deb S. K. The influence of microstructure on the electrochromic properties of Li_*x*_WO_3_ thin films: Part I. Ion diffusion and electrochromic properties. J. Mater. Res. 8, 2649–2656 (1993).

[b34] Julien C., Gorenstein A., Khelfa A., Guesdon J. P. & Ivanov I. Fabrication of V_2_O_5_ thin films and their electrochemical properties in lithium microbatteries. Mater. Res. Soc. Symp. Proc. 369, 639–647 (1995).

[b35] Navone C., Baddour-Hadjean R., Pereira-Ramos J. P. & Salot R. A kinetic study of electrochemical lithium insertion into oriented V_2_O_5_ thin films prepared by rf sputtering. Electrochim. Acta 53, 3329–3336 (2008).

[b36] Pyun S.-I. & Bae J.-S. Electrochemical lithium intercalation into vanadium pentoxide xerogel film electrode. J. Power Sources 68, 669–673 (1997).

[b37] Lu Y., Liu L., Mandler D. & Lee P. S. High switching speed and coloration efficiency of titanium-doped vanadium oxide thin film electrochromic devices. J. Mater. Chem. C 1, 7380–7386 (2013).

[b38] Tong Z. Q. *et al.* Improved electrochromic performance and lithium diffusion coefficient in three-dimensionally ordered macroporous V_2_O_5_ films. J. Mater. Chem. C 2, 3651–3658 (2014).

