# Supplementary material for: Novel morphology changes from 3D ordered macroporous structure to V2O5 nanofiber grassland and its application in electrochromism
Source: Sci Rep. 2015 Nov 18;5:16864. doi: 10.1038/srep16864 (PMC4649625; doi:10.1038/srep16864)
Supplement: Supplementary Information [file srep16864-s1.doc]

**Supporting information for:**

**Novel morphology changes from 3D ordered macroporous structure to V_2_O_5_ nanofiber grassland and its application in electrochromism**

Zhongqiu Tong^1^, Haiming Lv^1^, Xiang Zhang^1^, Haowei Yang^2^, Yanlong Tian^1^, Na Li^2^, Jiupeng Zhao^2^, Yao Li^1,*^

^1^ Center for Composite Materials and Structure, Harbin Institute of Technology, Harbin, 150001, China

^2^ School of Chemical Engineering and Technology, Harbin Institute of Technology, Harbin, 150001, China


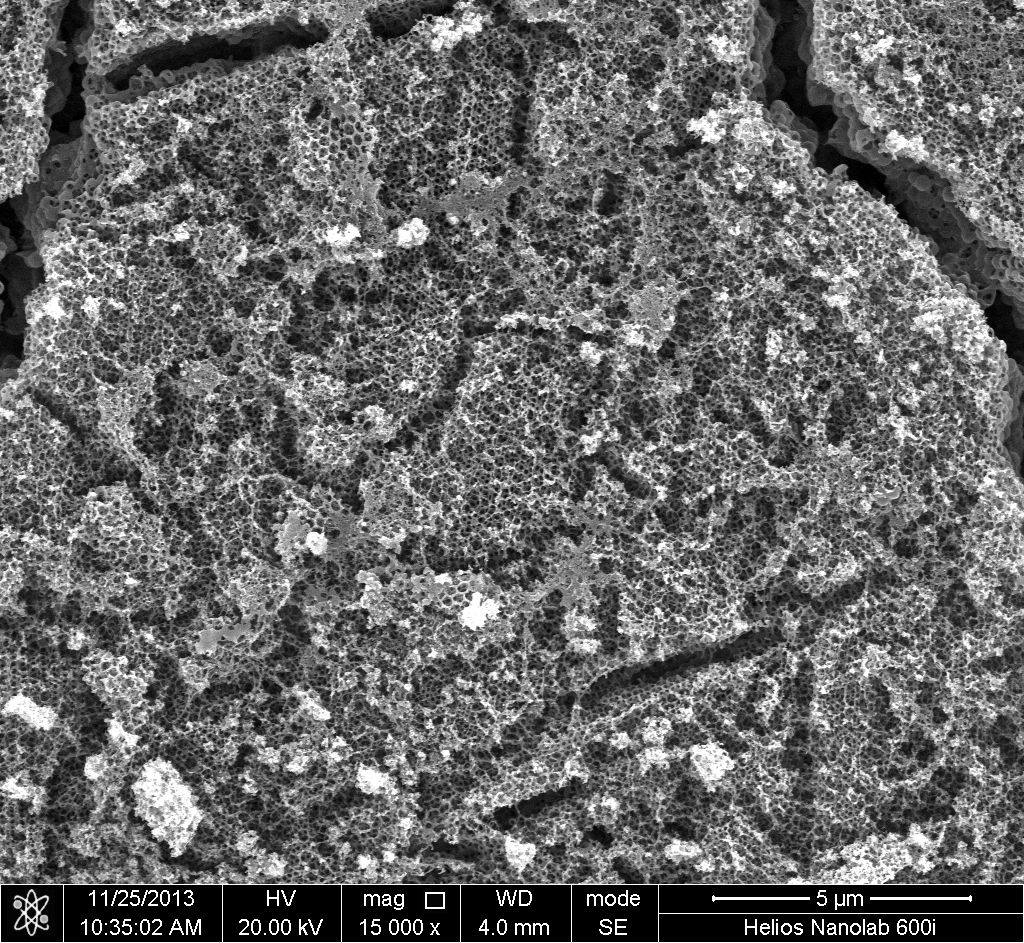


**Figure S1** Low-magnification SEM image of the amorphous 3DOM vanadia film.


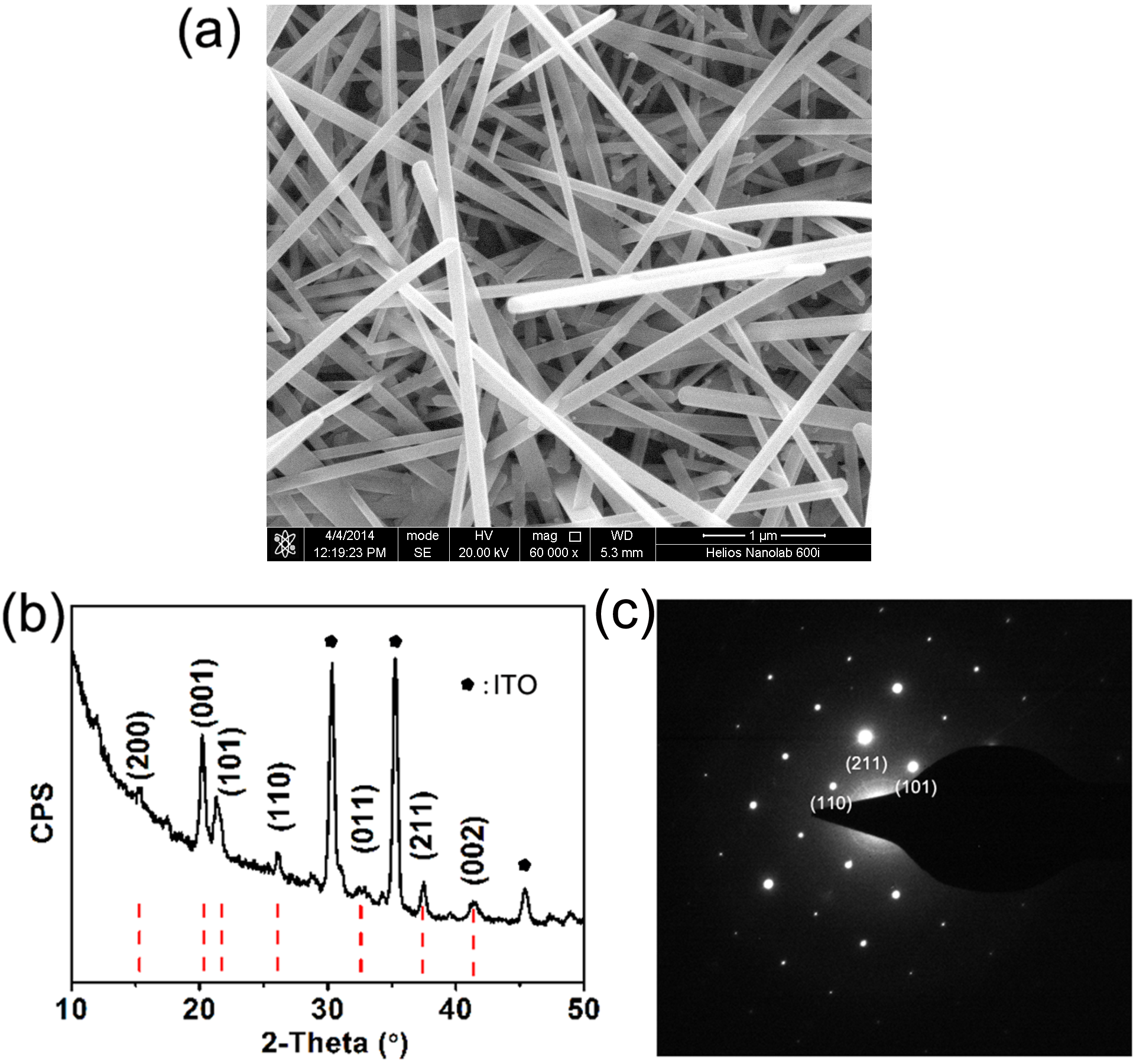


**Figure S2.** High-magnification SEM image (a) and XRD pattern (b) of the V_2_O_5_ nanofiber grassland. (c) SAED pattern taken from an α-V_2_O_5_ nanofiber.


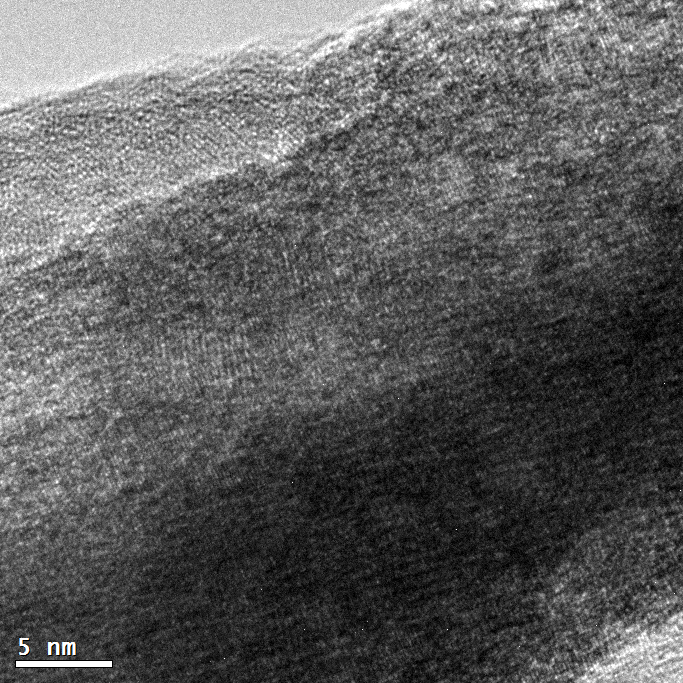


**Figure S3** HRTEM image shows the atom rearrangement process during the annealing treatment.


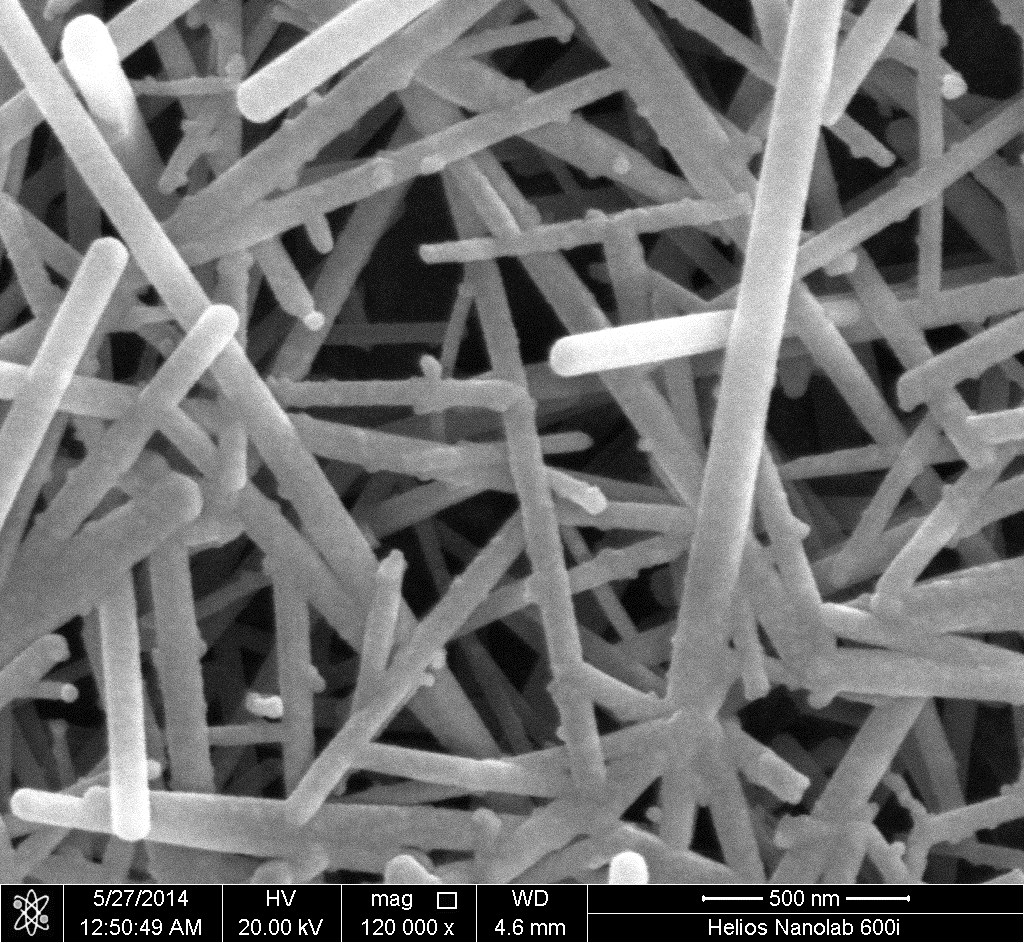


**Figure S4.** High-magnification SEM image of V_2_O_5_ nanofiber obtained after being annealed at 450 °C for 2 h.


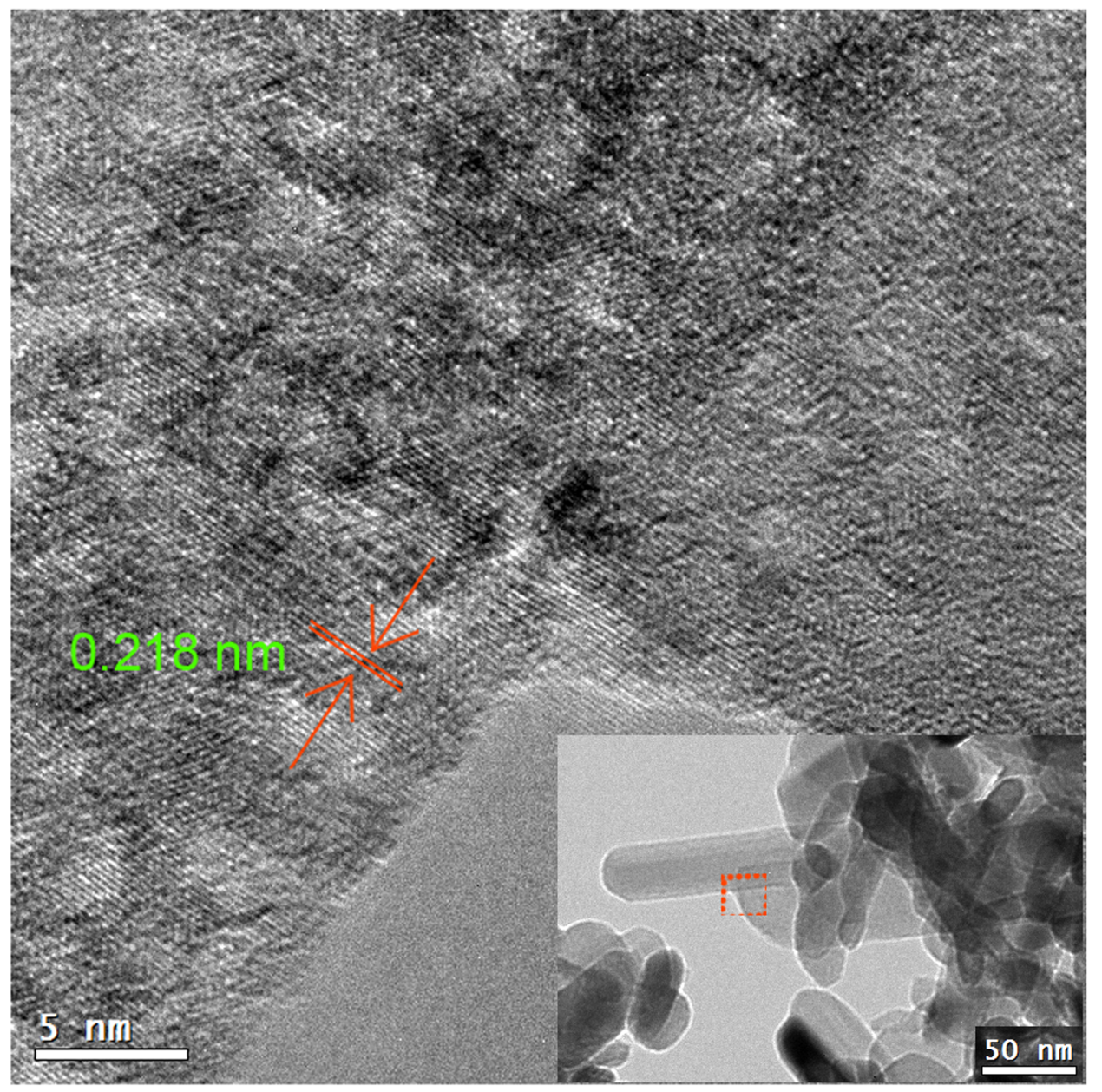


**Figure S5** HRTEM image shows the integrated single-crystal nanostructure stacked with two nanorods by shoulder to shoulder manner in the film annealed for 2 h..


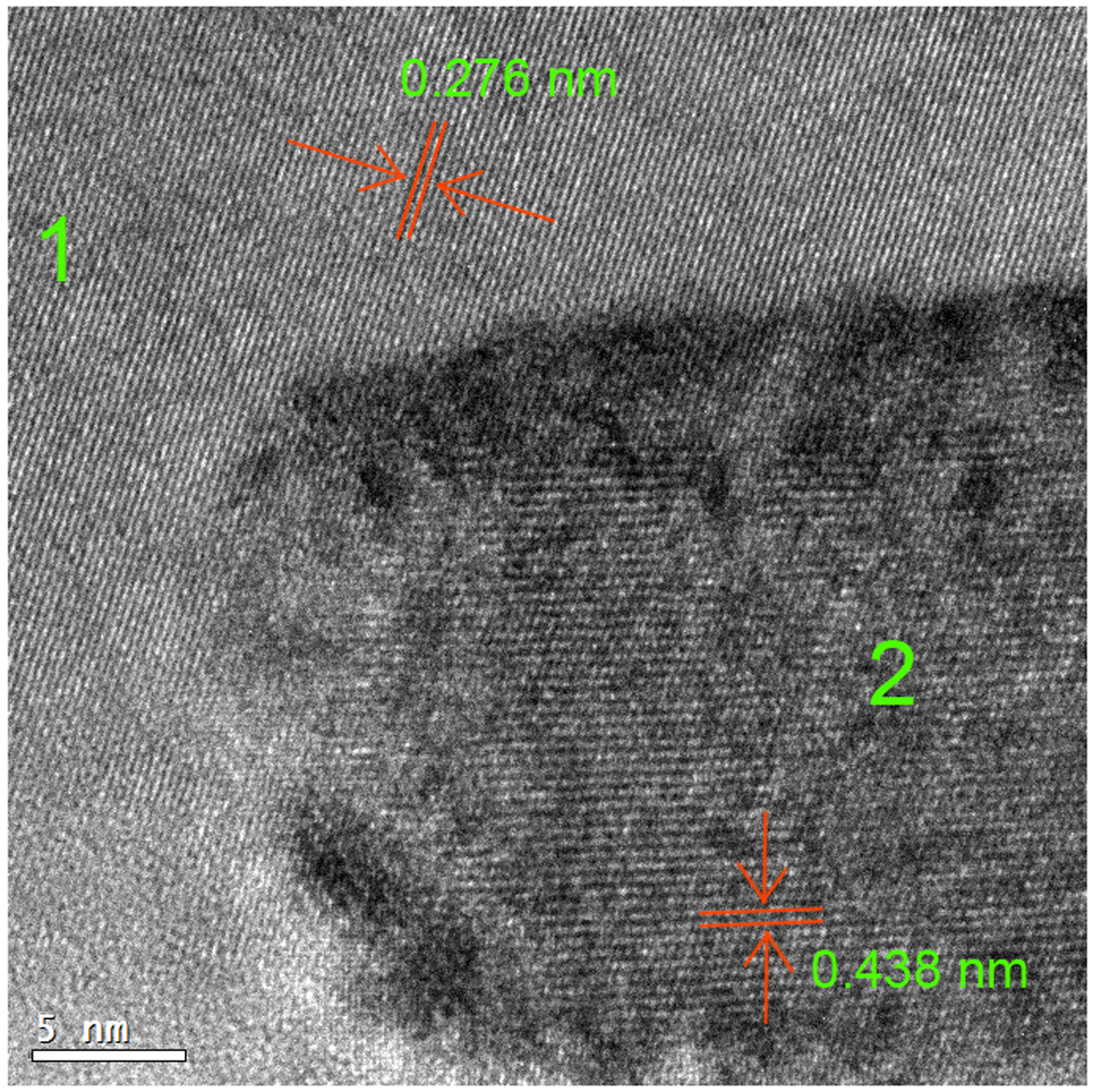


**Figure S6** HRTEM image shows the clear atom rearrangement process between the two crystallites in the film annealed for 2 h.


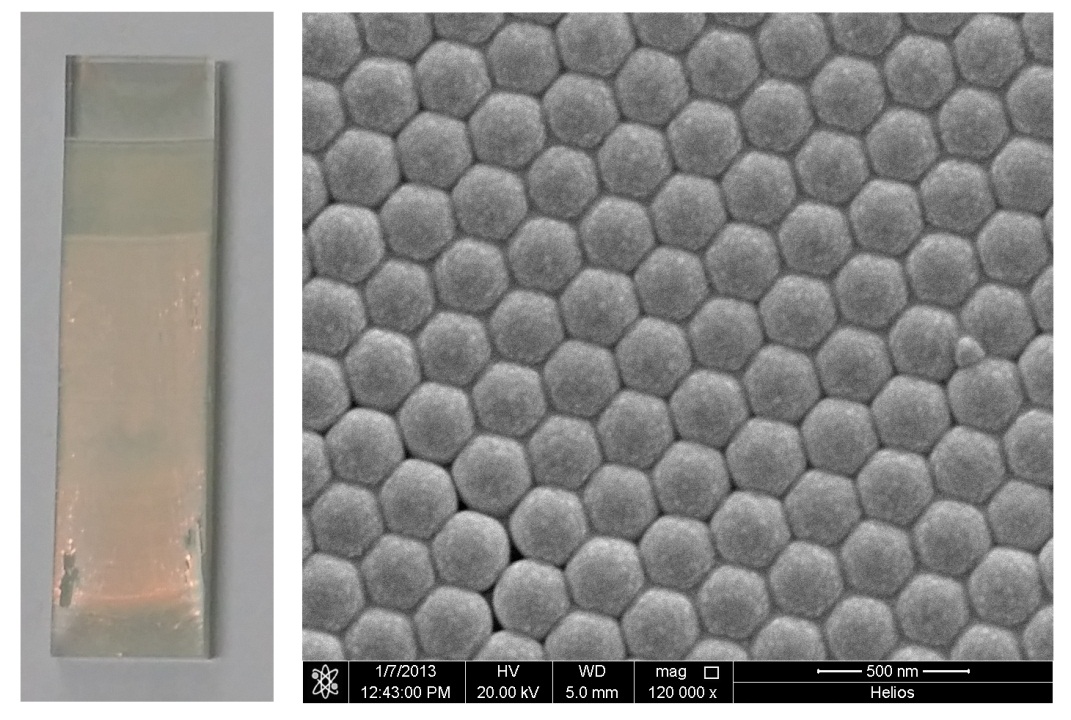


**Figure S7** Digital photograph and SEM image of the PS colloidal crystal.

**Table S1.** Comparison of the transmittance modulation of the V_2_O_5_ nanofiber grassland with that of other V_2_O_5_ films and nanostructures.

| Sample | Δ*T* at 460 nm (%) | Δ*T* at 1000 nm (%) | Reference |
| --- | --- | --- | --- |
| α-V_2_O_5_ nanofiber grassland | ca. 34 | ca. 25 | In this work |
| Reactive sputtering deposited V_2_O_5_ film | ca. 22 | ca. 15 | 1 |
| Ti-doped vanadia film by sol-gel method | ca.16 | ca. 17 | 2 |
| V_2_O_5_ film prepared by AACVD | ca.33 | ca.14 | 3 |
| V_2_O_5_ nanowire film | ca.25 | / | 4 |
| V_2_O_5_ nanobelt-membrane hybrid structure | ca.15 | ca. 60 at 900 nm | 5 |
| single-crystal  V_2_O_5_ nanorod arrays | ca.17 | ca. 26 at 900 nm | 6 |
| V_2_O_5_ film by sol-gel method | ca.13 | ca. 18 at 900 nm |
| V_2_O_5_ nanowire free-standing network | ca.12 | ca.34 | 7 |
| Gyroid-Structured V_2_O_5_ film | ca.22 | ca.40 | 8 |
| 3DOM vanadia film | ca.35 | ca.17 | 9 |

Note: AACVD is short for aerosol assisted chemical vapor deposition.

[1] Y.-S. Lin, C.-W. Tsai, *Surf. Coat. Technol.*, 2008, 202, 5641-5645.

[2] Y. X. Lu, L. Liu, D. Mandler, P. S. Lee, *J. Mater. Chem. C*, 2013, 1, 7380-7386.

[3] D. Vernardou, D. Louloudakis, N. Katsarakis, E. Koudoumas, I. I. Kazadojev, S. O’Brien, M. E. Pemble, I. M. Povey, *Sol. Energy Mater. Sol. Cells*, doi:10.1016/j.solmat.2014.12.002

[4] K.-C. Cheng, F.-R. Chen, J.-J. Kai, *Sol. Energy Mater. Sol. Cells*, 2006, 90, 1156-1165.

[5] W. B. Kang, C. Y. Yan, X. Wang, C. Y. Foo, A. W. M. Tan, K. J. Z. Chee, P. S. Lee, *J. Mater. Chem. C*, 2014, 2, 4727-4732.

[6] K. Takahashi, Y. Wang, G. Z. Cao, *Appl. Phys. Lett.*, 2005, 86, 053102.

[7] C. R. Xiong, A. E. Aliev, B. Gnade, K. J. Balkus, Jr., *ACS Nano*, 2008, 2, 293-301.

[8] M. R. J. Scherer, L. Li, P. M. S. Cunha, O. A. Scherman, U. Steiner, *Adv. Mater.,* 2012, 24, 1217-1221.

[9] Z. Q. Tong, H. W. Yang, L. Na, H. Y. Qu, X. Zhang, J. P. Zhao, Y. Li, *J. Mater. Chem. C,* 2015, 3, 3159-3166.

**Table S2**. The simulated *R*_e_ and *R*_ct_ values of the α-V_2_O_5_ nanofiber grassland under different polarized potentials

|  | α-V_2_O_5_ nanofiber grassland under different polarized potentials | | | | | |
| --- | --- | --- | --- | --- | --- | --- |
|  | 1 V (Ω) | 0.6 V (Ω) | 0.2 V (Ω) | -0.2 V (Ω) | -0.6 V (Ω) | -1 V (Ω) |
| *R*_e_ | 4.2 | 4.4 | 4.8 | 5.1 | 7.3 | 8.4 |
| *R*_ct_ | 59.1 | 60.0 | 65.2 | 68.7 | 98.8 | 136.1 |
